# Genome-Wide Association Study Using Whole-Genome Sequence Data for Fertility, Health Indicator, and Endoparasite Infection Traits in German Black Pied Cattle

**DOI:** 10.3390/genes12081163

**Published:** 2021-07-28

**Authors:** Manuel J. Wolf, Tong Yin, Guilherme B. Neumann, Paula Korkuć, Gudrun A. Brockmann, Sven König, Katharina May

**Affiliations:** 1 Institute of Animal Breeding and Genetics, Justus-Liebig—University of Gießen, 35390 Gießen, Germany; manuel.j.wolf@agrar.uni-giessen.de (M.J.W.); tong.yin@agrar.uni-giessen.de (T.Y.); sven.koenig@agrar.uni-giessen.de (S.K.); 2 Albrecht Daniel Thaer—Institute for Agricultural and Horticultural Sciences, Animal Breeding Biology and Molecular Genetics, Humboldt-Universität of Berlin, 10115 Berlin, Germany; guilherme.neumann@hu-berlin.de (G.B.N.); paula.korkuc@hu-berlin.de (P.K.); gudrun.brockmann@hu-berlin.de (G.A.B.)

**Keywords:** complex traits, candidate genes, dairy cows, dual-purpose cattle, endangered breed, endoparasite resistance, GWAS

## Abstract

This genome-wide association study (GWAS) aimed to identify sequence variants (SVs) and candidate genes associated with fertility and health in endangered German Black Pied cattle (DSN) based on whole-genome sequence (WGS) data. We used 304 sequenced DSN cattle for the imputation of 1797 genotyped DSN to WGS. The final dataset included 11,413,456 SVs of 1886 cows. Cow traits were calving-to-first service interval (CTFS), non-return after 56 days (NR56), somatic cell score (SCS), fat-to-protein ratio (FPR), and three pre-corrected endoparasite infection traits. We identified 40 SVs above the genome-wide significance and suggestive threshold associated with CTFS and NR56, and three important potential candidate genes (*ARHGAP21*, *MARCH11*, and *ZNF462*). For SCS, most associations were observed on BTA 25. The GWAS revealed 61 SVs, a cluster of 10 candidate genes on BTA 13, and 7 pathways for FPR, including key mediators involved in milk fat synthesis. The strongest associations for gastrointestinal nematode and *Dictyocaulus viviparus* infections were detected on BTA 8 and 24, respectively. For *Fasciola hepatica* infections, the strongest associated SVs were located on BTA 4 and 7. We detected 200 genes for endoparasite infection traits, related to 16 pathways involved in host immune response during infection.

## 1. Introduction

Rapid advances in sequencing technology have opened new opportunities for dairy cattle breeding. Whole-genome sequencing (WGS) and genotype imputation to whole-genome sequence genotypes is an effective method to identify genes and causal mutations for single-gene traits, genetic defects, and complex polygenic traits in cattle [1]. Compared to commonly used single nucleotide polymorphism (SNP) chip arrays, WGS data enable discovering both common and rare variants affecting complex polygenic traits in cattle [2]. Consideration of complete linkage information in WGS data plus the possible consideration of the full genetic variance may contribute to the identification of variants, which do not exceed the significance threshold in genome-wide association studies (GWAS) using commercial SNP panels [2,3]. As an outstanding step forward, using WGS data in GWAS enhances the discovery of causative mutations [4,5] and increases the power to map genes for low heritable functional traits in cattle [4,6,7].

Powerful gene mapping approaches for functional traits is of special importance for the conservation of local and endangered breeds, since these are less competitive to large cattle populations with regard to economically relevant traits [8,9]. Being predominantly kept in outdoor systems, local breeds are considered to be robust to harsh environmental conditions, displaying favorable adaptive trait characteristics such as heat stress or disease resistance [9,10,11]. The conservation of local endangered breeds is essential to offer solutions for future challenges in animal husbandry and to preserve genetic diversity [11]. In this regard, WGS data may include genomic markers which can be used to improve functional traits [12]. Moreover, compared with pedigree data, deep and dense genomic information as provided by WGS data improves inbreeding estimations and is powerful to identify deleterious variants in a population. Both aspects, i.e., controlling inbreeding and deleterious allele frequencies, are important mechanisms in genomic selection programs to maintain long-term selection response and genetic diversity [13].

With a small population size of ~2560 cattle, the German dual-purpose Black Pied cattle (DSN, German: Deutsches Schwarzbuntes Niederungsrind) is an endangered local breed in Germany [14]. The breed has a long breeding history in the German grassland region East Frisia, as well as in the German federal states of Lower Saxony and Brandenburg located in the eastern part of Germany [15,16]. DSN is considered the founder breed of the modern Holstein Friesian (HF) and characterized by higher fertility and milk protein and fat content compared to HF [10,17]. DSN cows are well adapted to grazing systems, and thus, 50% of all DSN cows in Germany are kept under organic pasture conditions [18]. However, grazing is associated with an increased risk for endoparasite infections [19].

Gastrointestinal nematodes (GIN), the bovine lungworm (*Dictyocaulus viviparus*), and the liver fluke (*Fasciola hepatica*) are the three most important helminthic species in pastured dairy cattle [20]. The annual estimated costs of these three helminth infections were 941 million euros in dairy cattle in Europe due to impairments in milk production and fertility [20]. Since anthelmintic treatment against endoparasite infections is restricted in organic dairy farming, there is a need to develop breeding strategies to improve cows’ resistance to helminthic parasites. In this regard, research has aimed to investigate the genetic background of resistance to endoparasite infections in dairy cattle since 2017 [21,22,23]. Surprisingly, in a cross-classified research design for selection line comparisons, May et al. [21] identified higher GIN and *D. viviparus* infection rates for DSN compared to HF. An ongoing GWAS [22] detected the strongest associations for GIN and *D. viviparus* infections on BTA 2, 5, 8, 15, 17, 21, and 24 in a population of 148 DSN cows with 700K imputed genotypes. For *F. hepatica*, associated significant SNP markers were identified on BTA 1, 7, and 28. The genes *ALCAM*, *CDH2*, *EGFR*, and *PHLPP1* were annotated as main candidates involved in immunological functions during endoparasite infections in DSN. As an extension to commercial SNP chip applications, GWAS based on WGS data is expected to unravel the genetic architecture of endoparasite resistance and further important functional traits in DSN much deeper. For example, Twomey et al. [7] applied a GWAS for endoparasite infection traits in Irish dairy and beef cattle and demonstrated that only 0 to 11% of all quantitative trait loci (QTL) from imputed WGS data were detected when using 50 K genotypes.

Further GWAS in DSN were based on commercial 50 K SNP chip panels with focus on milk production [24] and udder health traits [25]. These studies revealed candidate genes previously described in HF and other cattle breeds, raising the interest in identifying species-specific variants for functional traits in DSN with WGS data. Clinical mastitis is the most common disease in DSN with incidences up to 26% [25], raising the interest in improving somatic cell counts (SCC) in milk as an indicator for udder health in DSN via genomic selection. Jaeger [10] studied udder health indicator traits in DSN phenotypically and identified a strong detrimental impact of increased SCC on feed intake and rumination. Although well adapted to grazing systems, DSN are exposed to metabolic stress due to heat stress impact on pastures and associated mobilization of body reserve in response to restricted feed intake. The fat-to-protein ratio (FPR) is a valuable indicator for metabolic health (e.g., ketosis) and for metabolic stability in this regard [26,27]. Moreover, FPR might be a novel indicator trait for robustness in DSN, especially in the context of challenging heat stress environments in outdoor systems [28]. Due to their favorable grazing abilities, the conservation of DSN is financially supported by the German government. However, ongoing DSN breed competitiveness implies an optimization of the preventive health management in grazing and organic farming systems via genomic selection, especially to improve metabolic stability and resistance to infectious diseases.

Therefore, the present study aimed to identify genome-wide associations, potential candidate genes and pathways for female fertility, SCC as an indicator for udder health, FPR as an indicator for metabolic health and stability, and endoparasite infections in DSN based on WGS data. Providing genomic markers for fertility and functional health traits is important for the development of future genomic selection programs to preserve DSN and to improve the breed adaption towards pasture environments.

## 2. Materials and Methods

### 2.1. Cow Traits

Cow traits were available for 1886 DSN cows from eight dairy farms located in the German federal states of Brandenburg (five farms), Hesse (one farm), and Lower Saxony (two farms). The cows were born between 2005 and 2016. The first calving age ranged from 22 to 40 months (mean: 27.3 months). Fertility traits included calving-to-first service interval (CTFS) and the non-return after 56 days (NR56 = insemination success proved at day 56 after first insemination) in first parity cows. For the udder and metabolic health indicator traits SCC and FPR, we included the first test-day record between days in milk (DIM) 5 and 40 from the first parity. SCC was log-transformed into somatic cell score (= SCS = log_2_ (SCC/100,000) + 3) [29]. For fertility and health indicator traits, we excluded cows with a genetic breed (DSN) percentage lower than 90% according to our own developed algorithm to clearly differentiate between DSN and HF cows [30].

The endoparasite infection traits considered repeated measurements for fecal egg counts for GIN (FEC-GIN), fecal egg counts for *F. hepatica* (FEC-FH), and fecal larvae counts (FLC) for *D. viviparus* (FLC-DV). A modified McMaster technique [31] with a sensitivity of 25 eggs/g feces was used to determine FEC-GIN. For GIN, strongylid eggs were the predominant morphotype followed by *Strongyloides papillosus* and *Capillaria* spp. eggs. Fecal egg counts for *F. hepatica* were examined by the sedimentation technique using 10 g feces per sample. The fecal larvae count for *D. viviparus* was determined with the Baermann technique using 40 g feces per sample [32]. Endoparasite traits (FEC-GIN, FLC-DV, FEC-FH) were available for an initial dataset including 1166 untreated and pastured Black and White dairy cows (including DSN) examined in 2015 [21]. Using this initial dataset, endoparasite traits were pre-corrected for fixed effects via linear mixed models in the statistical software SAS (version 9.4 [33]) as described in May et al. [22]. In this regard, farm, parity, genetic line (DSN and other Holstein Friesian selection lines), season of parasitological examination, and lactation stage of cows were included as fixed effects. The pre-corrected phenotypes (residuals) for the three endoparasite traits (FEC-GIN, FEC-FH, and FLC-DV) from the linear mixed models are later denoted as RES-GIN, RES-FH, and RES-DV, respectively. Descriptive statistics for FEC-GIN, FLC-DV, and FEC-FH in the initial dataset of 1166 cows are presented in Table 1.

### 2.2. Genetic Architecture of Cow Traits

Genetic parameters were estimated using the software GCTA [34] for CTFS, NR56, SCS, and FPR considering the genomic relationship matrix (**G**) and applying linear mixed models as described in Section 2.5. for the GWAS. In this regard, the genomic relationship matrix was constructed in GCTA according to VanRaden [35]. The SNP-based heritabilities were 0.05 (±0.03) and 0.02 (±0.02) for CTFS and NR56, respectively. For SCS and FPR, the SNP-based heritabilities were 0.13 (±0.04) and 0.14 (±0.04), respectively. For endoparasite traits, we estimated heritabilities based on the pedigree matrix (**A**) using the initial dataset of 1166 cows [21]. The pedigree-based heritabilities were 0.06 (±0.04) for FEC-GIN, 0.05 (±0.04) for FEC-DV, and 0.33 (±0.06) for FEC-FH. 

### 2.3. Whole-Genome Sequencing and Imputation of 50K Genotypes

Whole-genome sequencing data were available for 304 DSN cattle, of which 47 were bulls (all available DSN sires used for artificial insemination) and 257 were cows from eight different herds, reflecting the whole phenotypic range for milk yield, milk composition, and reproduction traits in the DSN population. Sequencing was performed on the Illumina NovaSeq platform (Novogene Bioinformatics Technology Co., Ltd., Beijing, China) with 150 paired end reads and 15× coverage. Sequence read mapping, variant calling, and recalibration were performed following the 1000 Bull Genome (http://www.1000bullgenomes.com; accessed on 21 February 2020 guidelines. Lower quality sequence variants (SVs) were discarded by applying the machine learning method “variant quality score recalibration” in the Genome Analysis Toolkit (GATK, version 4.1.3.0 [36]) and using training and truth set variants provided by the 1000 Bull Genomes database. After, the dataset included 20,567,619 SVs (including 18.5 million SNPs and 2.0 million indels). SVs with a minor-allele frequency (MAF) <1% and a call rate < 95% were discarded. SVs with 5% of Mendelian inconsistences (i.e., opposing homozygotes detected from 156 sire-offspring pairs) were removed. Moreover, SVs with a quality by depth (QD) <10 and coverage <3000 were removed in case they were not predicted as high or moderate impact by the variant effect predictor (VEP) [37]. After quality filtering, the dataset contained 16,175,216 SVs from 304 DSN. This dataset provided a reference panel for the imputation of 1797 DSN genotyped with the BovineSNP50 (50 K) Bead Chip V2 (Illumina Inc., San Diego, CA, USA). The average numerator relationship between the 304 cattle from the reference panel and the 1797 genotyped cows for imputation was 6.7%. The inbreeding coefficients were 1.9% for the reference group and 2.1% for the imputation group. The imputation was performed in the software BEAGLE (version 5.1 [38]) based on a one-step imputation accuracy approach [39]. The average imputation accuracy was 97.04%. SVs with MAF <5%, linkage disequilibrium (*r*^2^) <0.5 (output from BEAGLE), and significant deviation from the Hardy–Weinberg equilibrium (HWE, *p* < 10^−6^) were discarded from the imputed dataset. Then, the 1797 imputed DSN genotypes were merged with the 304 sequenced DSN, resulting in a total number of 12,164,173 SVs (including 11,043,497 SNPs and 1,120,676 indels) from 2101 sequence level DSN genotypes.

### 2.4. Quality Control of Whole-Genome Sequence Genotypes

Imputed sequence-level genotypes and cow traits were merged, resulting in a total number of 1683 cows with phenotypes for fertility traits, 1638 cows with phenotypes for health and metabolic stability indicator traits, and 142 cows with phenotypes for pre-corrected endoparasite infection traits. Quality control of imputed WGS genotypes was performed within the three datasets in three consecutive runs in PLINK (version 1.9 [40]). SVs with MAF <5% and significant deviation from HWE (*p* < 10^−6^) or a call rate <95% were discarded. After quality control, 11,391,082 SVs (10,343,725 SNPs and 1,047,357 indels) remained for cows with phenotypes for CTFS and NR56, 11,413,456 SVs (10,363,951 SNPs and 1,049,505 indels) remained for cows with phenotypes for SCS and FPR, and 10,595,540 SVs (9,624,332 SNPs and 971,208 indels) remained for cows with pre-corrected phenotypes (residuals) for endoparasite infection traits (RES-GIN, RES-FH and RES-DV). Descriptive statistics for all traits of cows with imputed sequence level genotypes after quality control is shown in Table 1.

### 2.5. Genome-Wide Association Analyses

We applied a single marker linear mixed model in the software package GCTA [34] to identify genome-wide associations for fertility traits (CTFS, NR56), health and metabolic stability indicator traits (SCS, FPR), and pre-corrected endoparasite infection traits (RES-GIN, RES-FH, RES-DV). All GWAS were performed as MLMA-LOCO “leaving one chromosome out” analysis for large datasets using the --mlma and --mlma-subtract-grm options in GCTA. The statistical model for testing single-locus effects was defined as follows:

y = Xβ + Zu + Ss + e

where y = vector including records for CTFS, NR56, SCS, FPR, RES-GIN, RES-FH, and RES-DV; β = vector of fixed effects (farm, calving year, calving month, and a linear regression on age at first calving for CTFS and NR56; farm, test-day year-season, a linear regression on DIM, and a linear regression on fat percentage for SCS; farm, test-day year-season, and a linear regression on age at first calving for FPR); u = vector of polygenic effects with u* ~ N* (0, *Gσ^2^_u_*), with G denoting the genetic similarity matrix among individuals, and *σ^2^_u_* the polygenic variance; s = vector for marker effects; e = vector of random residuals; and X, Z, and S were incidence matrices for β, u, and s, respectively.

For model quality, we checked quantile-quantile (Q-Q) plots and calculated the genomic inflation factor λ for each association analysis. We applied a Bonferroni correction to account for multiple testing. Since traditional Bonferroni correction (i.e., relating the genome-wide significance threshold of 0.05 to the total number of markers) tends to produce many false-negative associations [41], we did not use the total number of SVs but estimated the effective number of independent SVs (n_eff_). To calculate n_eff_, one sequence variant (SV) of a SV pair in LD with *r*^2^ >0.5 was excluded using the --indep-pairwise option in PLINK [40]. A window size of 5000 SVs was chosen, which was shifted in an interval of 500 SVs. The adjusted Bonferroni-corrected genome-wide significance threshold based on n_eff_ (*p* = 0.05/n_eff_) was *p*Bonf = 1.60 × 10^−7^ (0.05/312,502) for CTFS and NR56, *p*Bonf = 1.56 × 10^−7^ (0.05/319,686) for SCS and FPR, and *p*Bonf = 1.22 × 10^−7^ (0.05/409,481) for RES-GIN, RES-FH, and RES-DV. In addition, we considered a less conservative suggestive significance threshold (*p*Sug = 1/n_eff_). The suggestive significance threshold was *p*Sug = 3.20 × 10^−6^ (1/312,502) for CTFS and NR56, *p*Sug = 3.13 × 10^−6^ (1/319,686) for SCS and FPR, and *p*Sug = 2.44 × 10^−6^ (1/409,481) for RES-GIN, RES-FH, and RES-DV, respectively.

### 2.6. Candidate Gene Annotations and Pathway Analyses

We applied the biomaRt R package [42,43] from Bioconductor to retrieve ‘rs accession numbers’ of associated SVs using the getBM() function. Potential candidate genes were queried and assigned to the associated SVs using the current gene annotations from ENSEMBL (release 104) [44] based on the *Bos taurus* ARS-UCD1.2 genome assembly [45]. A gene was considered as a candidate gene if at least one SV above *p*Sug was positioned in the respective gene and/or within 100 kb up- and downstream of the respective candidate gene. Physiological functions and positions of potential candidate genes were manually reviewed in the ENSEMBL and KEGG [46] databases. In addition, the identified potential candidate genes were manually submitted to the DAVID (version 6.8 [47]) and KEGG pathway databases for pathway analysis.

## 3. Results

### 3.1. Female Fertility Traits

#### 3.1.1. Calving-to-First Service Interval

**Figure 1:**
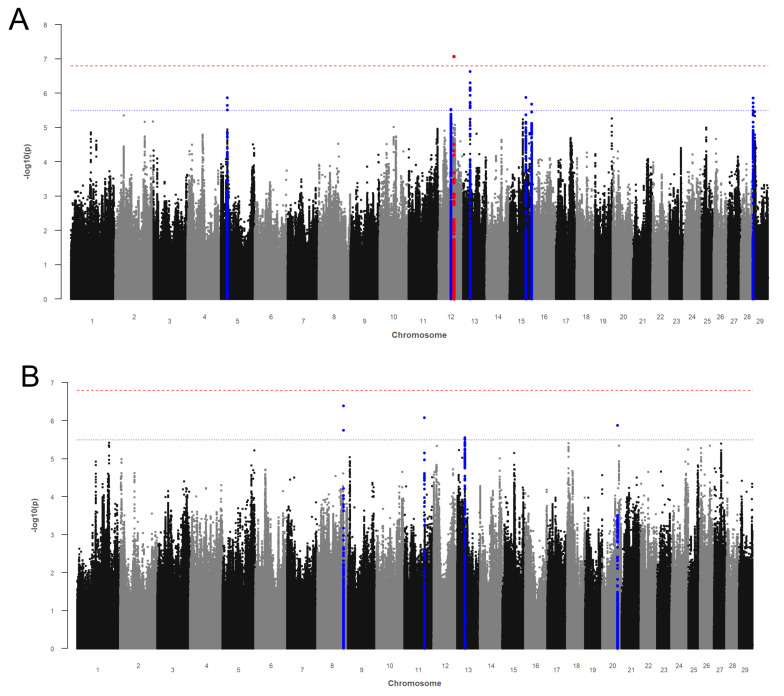
Manhattan plots for −log10 *p*-values of marker effects for (**A**) calving-to-first service interval (CTFS) and (**B**) non-return 56 (NR56). Markers above the genome-wide significance threshold *p*Bonf (red dashed line) are highlighted in red and markers above the suggestive significance threshold *p*Sug (blue dashed line) are highlighted in blue. Markers within a distance of 125 kb up- and downstream of the significantly or suggestively associated SVs are highlighted in red or blue, respectively.

The Manhattan plot from the GWAS with CTFS is given in Figure 1A. The genomic inflation factor λ was 1.041. We identified 33 SNPs above *p*Sug on BTA 5, 12, 13, 15, and 28 (Table S1). In addition, one SNP (rs380946888) on BTA 12 surpassed the genome-wide significance threshold *p*Bonf. The highest number of associations was identified on BTA 13 (*n* = 15) and on BTA 28 (*n* = 10). The associated markers were annotated to six potential candidate genes on BTA 12, 13, 15, and 28 (Table 2). On BTA 12, we identified the Kelch-like family member 1 (*KLHL1*) gene. The highest association (*p* = 2.25 × 10^−7^) on BTA 13 was found within the Rho GTPase activating protein 21 (*ARHGAP21*) gene with five SNPs located in the gene and nine SNPs and one indel close to the gene (Table 2). Further annotated genes were the lin-7 homolog C (*LIN7C*) gene, *ENSBTAG00000052005*, and the olfactory receptor family 5 subfamily member 5 (*OR5BE5*) gene on BTA 15. All associated variants on BTA 28 are located in the choline O-acetyltransferase (*CHAT*) gene.

#### 3.1.2. Non-Return after Day 56

Figure 1B shows the Manhattan plot from the GWAS with NR56. The genomic inflation factor λ was 1.014. The GWAS revealed six SVs (five SNPs and one indel) above *p*Sug on BTA 8, 11, 13, and 20 for NR56 (Table S1). The associated markers were annotated to three potential candidate genes (Table 2). The SNPs rs110809463 and rs135364419 on BTA 8 are located in the zinc finger protein 462 (*ZNF462*) gene. The SNP rs383197946 on BTA 11 is located in the EFR3 homolog B (*EFR3B*) gene. The SNP rs207515592 on BTA 20 is closely related to the membrane associated ring-CH-type finger 11 (*MARCH11*) gene.

### 3.2. Health Indicator Traits

#### 3.2.1. Somatic Cell Score

**Figure 2:**
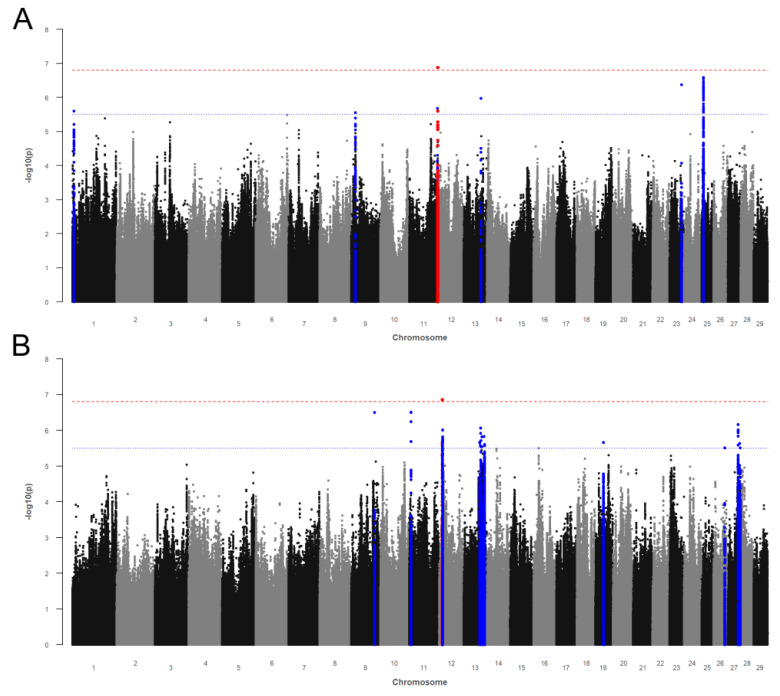
Manhattan plots for −log10 *p*-values of marker effects for (**A**) somatic cell score (SCS) and (**B**) fat-to-protein ratio (FPR). Markers above the genome-wide significance threshold *p*Bonf (red dashed line) are highlighted in red and markers above the suggestive significance threshold *p*Sug (blue dashed line) are highlighted in blue. Markers within a distance of 125 kb up- and downstream of the significantly or suggestively associated SVs are highlighted in red or blue, respectively.

The Manhattan plot from the GWAS with SCS is presented in Figure 2A. The genomic inflation factor λ was 1.072. The GWAS revealed one SNP (rs137783421) above *p*Bonf on BTA 11 and 53 SNPs above *p*Sug on BTA 1, 9, 11, 13, 23, and 25 (Table S2). The majority of associations (*n* = 47) was found on BTA 25. We identified nine potential candidate genes for SCS (Table 3). The claudin 8 (*CLDN8*) gene on BTA 1 was annotated to the leukocyte transendothelial migration pathway and to the cell adhesion molecules pathway, playing a major role in gene expressions during *Escherichia coli*-induced mastitis in dairy cows (Table 4). One marker on BTA 9 was located in the SUMO specific peptidase 6 (*SENP6*) gene. The SNP rs137783421 (maximum association for SCS) on BTA 11 is located in the Rap guanine nucleotide exchange factor 1 (*RAPGEF1*) gene. The RAN binding protein 9 (*RANBP9*) gene on BTA 23 was annotated to the SNP rs876215027. All genomic associations on BTA 25 (*n* = 47; bp: 6,629,679–6,656,058) are located in or close to the RNA binding fox-1 homolog 1 (*RBFOX1*) gene.

#### 3.2.2. Fat-to-Protein Ratio

The Manhattan plot from the GWAS for FPR is presented in Figure 2B. The genomic inflation factor λ was 1.088. The GWAS revealed one SNP (rs439994366) above *p*Bonf on BTA 12 and 60 SVs (54 SNPs and six indels) above *p*Sug on BTA 9, 11, 12, 13, 19, 26, and 27 (Table S2). The associated markers were annotated to 19 potential candidate genes (Table 3). The SNP rs439994366 is located in the Von Willebrand factor A domain containing 8 (*VWA8*) gene on BTA 12, with 15 SVs (thereof two indels) located in the gene and one further SNP at a close distance. The region between 58.4 and 75.3 Mb on BTA 13 harbored a cluster of ten potential candidate genes. On BTA 27, we identified the GINS complex subunit 4 (*GINS4*) gene, the glycerol-3-phosphatase acetyltransferase 4 (*GPAT4*) gene, and the thyroid hormone receptor β (*THRB*) gene.

The glutamate metabotropic receptor 1 (*GRM1*) gene is related to the calcium signaling, neuroactive ligand-receptor interaction, and phospholipase D signaling pathway, being associated with milk yield in dairy cattle (Table 4). Furthermore, the angiopoietin 4 (*ANGPT4*) gene and the thyroid hormone receptor β (*THRB*) gene are located in four pathways previously described for milk fat or protein synthesis in dairy cows (Table 4). 

### 3.3. Endoparasite Infection Traits

#### 3.3.1. Gastrointestinal Nematode Infections

**Figure 3:**
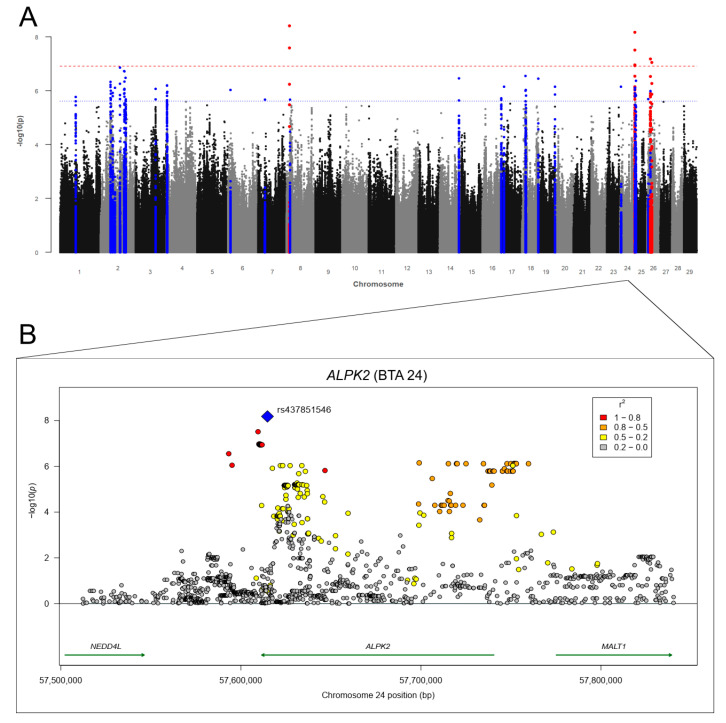
(**A**) Manhattan plot for −log10 *p*-values of marker effects for residuals of fecal egg counts of gastrointestinal nematodes (RES-GIN). Markers above the genome-wide significance threshold *p*Bonf (red dashed line) are highlighted in red and markers above the suggestive significance threshold *p*Sug (blue dashed line) are highlighted in blue. Markers within a distance of 125 kb up- and downstream of the significantly or suggestively associated SVs are highlighted in red or blue, respectively. (**B**) Regional association plot for the α kinase 2 (*ALPK2*) gene on BTA 24 with corresponding flanking regions (+/−100,000 bp). The SNP rs437851546 (blue square) was the highest associated SNP (*p* = 6.63 × 10^−9^) for RES-GIN on BTA 24. Circles show GWAS *p*-values, with different colors indicating linkage disequilibrium (LD): red: LD 0.8 to 1.0, orange: LD 0.5 to 0.8. yellow: LD 0.2 to 0.5, gray: LD 0.0 to 0.2. Genes with a green arrow pointing to the right are located on the forward strand, genes with an arrow pointing to the left are located on the reverse strand.

The associations between each SV and RES-GIN are shown in the Manhattan plot in Figure 3A. The genomic inflation factor λ was 1.019. We identified 47 SVs (thereof one indel) above *p*Bonf and 270 SNPs and 30 indels above *p*Sug on 14 chromosomes associated with RES-GIN, respectively (Table S3). Most significantly and suggestively associations were identified on BTA 2 (*n* = 165) and on BTA 24 (*n* = 89). The strongest associations were identified on BTA 8 with seven markers surpassing *p*Bonf. Additionally, 29 SNPs and one indel on BTA 24 and 10 SNPs on BTA 26 surpassed *p*Bonf. The majority of associations surpassing *p*Sug was found on BTA 2 (*n* = 165) and BTA 24 (*n* = 59) (Table S3). Significantly and suggestively associated SVs were annotated to 46 potential candidate genes (Table 5; Table S4). The most interesting candidate genes with at least 10 SVs located in the gene were the contactin-associated protein-like 5 (*CNTNAP5*) gene and the microtubule-associated protein 2 (*MAP2*) gene on BTA 2. On BTA 24, we identified 23 SVs (22 SNPs and one indel) located in the α kinase 2 (*ALPK2*) gene and 58 further SVs (thereof one indel) in close distance (<19.2 kb) to *ALPK2* (Figure 3B). Both genes NEDD4 like E3 ubiquitin protein ligase (*NEDD4L*) and MALT1 paracaspase (*MALT1*) flank *APLK2*, forming an association cluster (bp: 57,593,275–57,876,049) with a total number of 79 associated SNPs and three associated indels (Figure 3B). In addition, we detected ten SNPs in the cartilage acidic protein 1 (*CRTAC1*) gene on BTA 26. 

As shown in Table 4, the gene activin receptor type 1C (*ACVR1C*) on BTA 2 is related to the cytokine–cytokine interaction pathway and to the TGF-ß signaling pathway, both involved in host immune response mechanisms. The adenylate cyclase 1 (*ADCY1*) on BTA 4 is part of the chemokine signaling and estrogen signaling pathways, previously identified for GIN infections in cattle (Table 4). Further pathways involved in host immune response were annotated to the *MALT1* and PH domain leucine rich repeat protein phosphatase 1 (*PHLPP1*) gene on BTA 24. These pathways include the B cell receptor signaling pathway, C-type lectin receptor signaling pathway, NF-kappa B signaling pathway, PI3K-Akt signaling pathway, and the T cell receptor signaling pathway (Table 4).

#### 3.3.2. Liver Fluke (*Fasciola hepatica*) Infections

**Figure 4:**
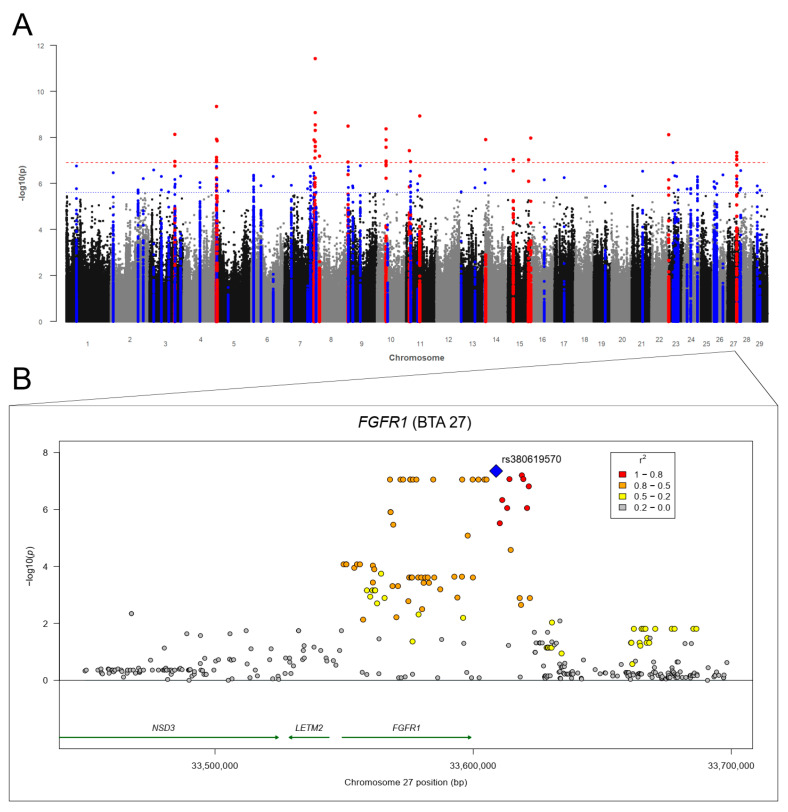
(**A**) Manhattan plot for −log10 *p*-values of marker effects for residuals of fecal egg counts of *Fasciola hepatica* (RES-FH); markers above the genome-wide significance threshold *p*Bonf (red dashed line) are highlighted in red and markers above the suggestive significance threshold *p*Sug (blue dashed line) are highlighted in blue. Markers within a distance of 125 kb up- and downstream of significantly or suggestively associated SVs are highlighted in red or blue, respectively. (**B**) Regional association plot for the fibroblast growth factor receptor 1 (*FGFR1*) gene on BTA 27 with corresponding flanking regions (+/−100,000 bp). The SNP rs380619570 (blue square) was the highest associated SNP (*p* = 4.48 × 10^−8^) for RES-FH on BTA 27. Circles show GWAS *p*-values, with different colors indicating linkage disequilibrium (LD): red: LD 0.8 to 1.0, orange: LD 0.5 to 0.8. yellow: LD 0.2 to 0.5, gray: LD 0.0 to 0.2. Genes with a green arrow pointing to the right are located on the forward strand, genes with an arrow pointing to the left are located on the reverse strand.

The Manhattan plot for the GWAS with RES-FH is shown in Figure 4A. The genomic inflation factor λ was 1.025. We identified 80 SVs (69 SNP and 11 indels) above *p*Bonf on BTA 3, 4, 7, 8, 9, 10, 11, 14, 15, 23, and 27 (Table S3). The strongest association (*p* = 3.66 × 10^−12^) was detected on BTA 7 (bp: 109,376,871). The GWAS revealed 224 further SVs (200 SNPs and 24 indels) above *p*Sug on all BTA despite of BTA 12, 18, 20, 22, and 25 (Table S3). The highest number of associations (above *p*Bonf and *p*Sug) were identified on BTA 4 (*n* = 51) and BTA 7 (*n* = 64). We identified 62 potential candidate genes for RES-FH (Table 5; Table S5). The region between 117.0 and 119.4 Mb on BTA 4 harbored a cluster of eight potential candidate genes (Table S5). The KIAA0825 (*KIAA0825*) gene and the mannosidase α class 2A member 1 (*MAN2A1*) gene were the most interesting candidate genes on BTA 7 with six SNPs in the *KIAA0825* gene and five SNPs in the *MAN2A1* gene plus five flanking SNPs, respectively. On BTA 27, 11 significantly associated SVs (thereof one indel) are located in the fibroblast growth factor receptor 1 (*FGFR1*) gene, and 12 significantly associated SVs (thereof on indel) are flanking around the gene (Figure 4B).

The identified candidate genes were annotated to 12 pathways potentially involved in host-parasite interactions (Table 4). The phospholipase C β 1 (*PLCB1*) gene on BTA 13 (three SNPs located in the gene) was assigned to four biological pathways potentially involved in host–*Fasciola hepatica* interactions (Table 4). We identified two suggestively associated SNPs in the protein tyrosine kinase 2 (*PTK2*) gene in BTA 14, which is part of the chemokine signaling, leukocyte transendothelial migration, and natural killer cell mediated cytotoxicity pathways (Table 4). The four genes interleukin 21 (*IL21*), prolactin-related protein VII (*PRP-VII*), prolactin-related protein IX (*PRP9*) and SMAD family member 4 (*SMAD4*) were related to the cytokine–cytokine receptor interaction, JAK-STAT signaling, PI3K-Akt signaling, TGF-β signaling, and Th17 cell differentiation pathways, respectively (Table 4).

#### 3.3.3. Bovine Lungworm (*Dictyocaulus viviparus*) Infections

**Figure 5:**
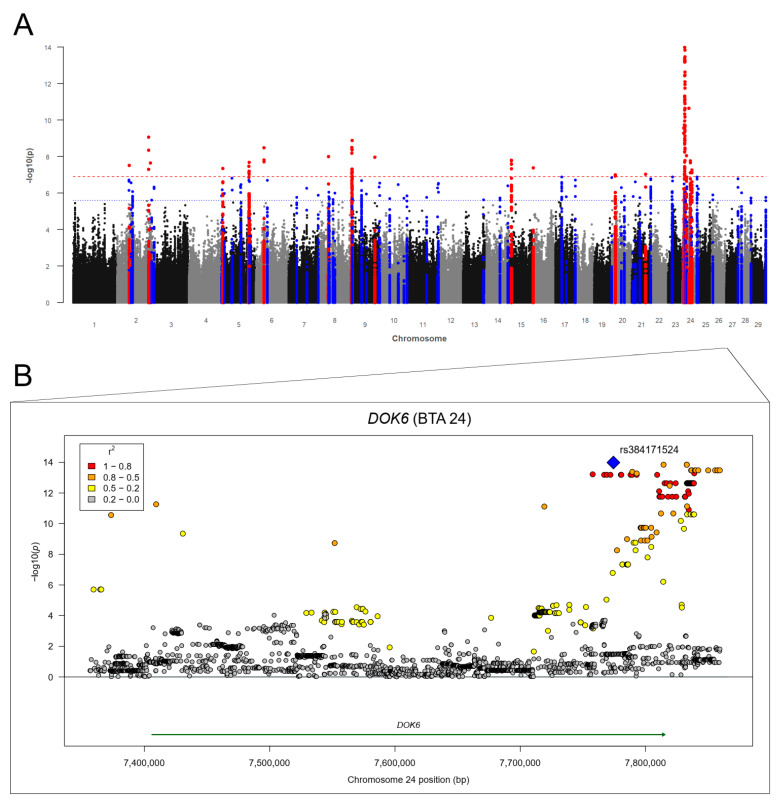
(**A**) Manhattan plot for −log10 *p*-values of marker effects for residuals for fecal larvae counts of *Dictyocaulus viviparus* (RES-DV); markers above the genome-wide significance threshold *p*Bonf (red dashed line) are highlighted in red and markers above the suggestive significance threshold *p*Sug (blue dashed line) are highlighted in blue. Markers within a distance of 125 kb up- and downstream of the significantly or suggestively associated SVs are highlighted in red or blue, respectively. (**B**) Regional association plot for the docking protein 6 (*DOK6*) gene on BTA 24 with corresponding flanking regions (+/−100,000 bp). The SNP rs384171524 (blue square) was the highest associated SNP (*p* = 1.04 × 10^−14^) for RES-DV. Circles show GWAS *p*-values, with different colors indicating linkage disequilibrium (LD): red: LD 0.8 to 1.0, orange: LD 0.5 to 0.8. yellow: LD 0.2 to 0.5, gray: LD 0.0 to 0.2.

The Manhattan plot for the GWAS with RES-DV is shown in Figure 5A. The genomic inflation factor λ was 1.064. We identified 332 SVs (311 SNPs and 21 indels) above *p*Bonf on BTA 2, 5, 6, 9, 15, 20, and 24 (Table S3). The most (*n* = 208) and strongest associations above *p*Bonf were identified on BTA 24. The GWAS revealed 823 further SVs (761 SNPs and 62 indels) above *p*Sug on all BTA except for BTA 1, 12, 18, 19, 22, and 25. The highest number of associations above *p*Sug were detected on BTA 9 (*n* = 231) and on BTA 24 (*n* = 214). We identified 92 potential candidate genes for RES-DV (Table 5; Table S6). The region between 94.9 and 97.7 Mb on BTA 5 includes a cluster of seven potential candidate genes (Table S6). The genes including the largest number of associations within this cluster were the Rho GDP dissociation inhibitor β (*ARHGDIB*) gene, the glutamate ionotropic receptor NMDA type subunit 2B (*GRIN2B*) gene, and the epithelial membrane protein 1 (*EMP1*) gene. We identified the contactin 5 (*CNTN5*) gene on BTA 15 with 47 significantly associated SVs (thereof four indels) located in the gene and 56 associated SVs (thereof three indels) in the flanking region of the gene. The region between 68.2 and 68.4 Mb on BTA 21 includes a cluster of five potential candidate genes. The strongest association within this cluster (*p* = 1.62 × 10^−7^; bp 68,299,895) was located in the kinesin light chain 1 (*KLC1*) gene, with a total number of 59 SVs (49 SNPs and 10 indels) located in the gene (Table S6). On BTA 24, we identified a cluster of five genes including a large number of associations in the region between 7.0 and 8.4 Mb. These genes include the suppressor of cytokine signaling 6 (*SOCS6*) gene, the rotatin (*RTTN*) gene, the CD226 molecule (*CD226*) gene, the docking protein 6 (*DOK6*) gene, and the coiled-col domain containing 102B (*CCDC102B*) gene. Within this cluster, we identified the largest number of associations for *DOK6* (Figure 5B). A further potential candidate gene was laminin subunit α 3 (*LAMA3*) with 34 SVs (thereof two indels) located in the gene.

The genes *SOCS6*, *CD226*, and *LAMA3* were related to the JAK-STAT signaling, cell adhesion molecules, and PI3K-Akt signaling pathways, respectively (Table 4). The bone morphogenetic protein receptor type 1B (*BMPR1*) gene on BTA 6 was annotated to the cytokine–cytokine receptor interaction and to the TGF-β signaling pathway, which are involved in the secretion and up- and downregulation of cytokines during helminth infections (Table 4). In addition, we identified the JAK-STAT signaling pathway and the PI3K-Akt signaling pathway for the cyclin D3 (*CCND3*) gene.

## 4. Discussion

This is the first study investigating genome-wide associations and candidate genes for functional traits in the local endangered DSN breed based on imputed WGS data. In our study, we used a quite large reference population of 304 sequenced DSN for imputation, resulting in a high imputation accuracy of 97.04%. Brondum et al. [67] and Mao et al. [68] demonstrated higher imputation accuracies for WGS data when using multi-breed compared to single-breed reference populations. Lower imputation accuracies of up to 95% were achieved in cattle studies using multi-breed reference panels with 242 to 1577 sequenced cattle [5,67,69]. Korkuć et al. [39] compared different imputation strategies in DSN and suggested a large reference panel of the same breed instead of a multi-breed (DSN together with HF) approach. Achieving an imputation accuracy above 97%, we expected a high power in our GWAS to identify genomic loci associated with complex functional traits in DSN.

We focused on fertility, udder health, metabolic health and stability indicator traits, and endoparasite infection traits since these are highly relevant for the conservation of the breed [10,24]. For CTFS, we identified SVs on BTA 12, 13, 15, and 28 with the largest number of associations on BTA 13. In Holstein cows, genomic loci for CTFS were detected on BTA 13, too [70], but associated regions on BTA 13 did not overlap with our findings. We identified *ARHGAP21* on BTA 13 as one main candidate gene for CTFS with five SNPs located in the gene or within a region up to 42.3 kb downstream of the gene. The *ARHGAP21* gene was reported to be involved in post-partum anestrus in tropical beef cattle [71]. Rosa et al. [72] demonstrated in a mouse model that *ARHGAP21* is involved in insulin secretion. Insulin plays a major role in the regulation of energy balance and reproductive functions in dairy cattle [73]. Gong et al. [74] showed that diets with higher insulin levels significantly reduced the intervals from calving to first ovulation in dairy cows. Thus, *ARHGAP21* might impact CTFS by regulating important hormone functions in dairy cattle. For NR56, our GWAS revealed significant SVs on BTA 8, 11, 13, and 20. Holmberg and Andersson-Eklund et al. [75] identified SNPs on BTA 11 and on BTA 20 associated with NR56 in Swedish dairy cows. We identified three genes for NR56, of which two were previously reported to be involved in cattle female fertility. The *ZNF462* gene was identified as a transcription factor involved in fertility in Brangus heifers [76]. Moore et al. [77] showed reduced expressions of *ZNF462* in the corpus luteum of low fertility HF cows compared to the high fertility control group. Kiser et al. [78] described *MARCH11* on BTA 20 as a top locus for conception rate at first service and repeated artificial insemination in Holstein heifers. Furthermore, *MARCH11* was recently associated with endometriosis in HF cows in our studies [79]. Hence, our detected genes influencing fertility in DSN may have similar functions for fertility in other cattle breeds. This observation is in accordance with Korkuć et al. [24], who found a high overlap of genomic regions in DSN and HF for milk production traits, although they identified no common genes for DSN and HF. Although we identified genes for CTFS and NR56 in DSN which were previously described in other breeds, the same gene can be differentially expressed in different breeds, which might phenotypically explain improved fertility in DSN. For example, Timperio et al. [80] showed transcriptomic level disparities in liver tissues in two closely related *Bos taurus* breeds, which contribute to large physiological differences. Moreover, Lehnert et al. [81] demonstrated that gene expression levels for growth-related genes can differ in different breeds, although the genes are involved in growth in cattle generally.

For FPR, we detected a cluster of ten closely related genes on BTA 13. This finding is in contrast to the GWAS by Korkuć et al. [24], where no SNP reached the significance threshold on BTA 13 for milk fat and protein traits in a population of 1816 50K genotyped DSN. However, similar to our findings, the authors detected SVs on BTA 9, 11, 12, and 27 associated with milk fat content or milk fat yield in DSN. Interestingly, Klein et al. [27] identified similar associations for FPR in Holstein cows on BTA 9, 13, 14, and 27, indicating a strong genomic relatedness of HF and DSN. The *DGAT1* gene on BTA14 was highly associated with FPR in Holstein cows [27]. Furthermore, Jaeger [10] identified *DGAT1* for fat content in a multi-breed GWAS including a quite large number of DSN cows. However, although genomic relatedness between DSN and HF is high, we detected no associations in the *DGAT1* gene for FPR in DSN, which coincides with the observation by Korkuć et al. [24] for other milk production traits in DSN. We identified the glycerol-3-phosphatase acyltranserase 4 (*GPAT4*) gene on BTA 27 as a potential candidate gene for FPR. The *GPAT4* gene is well described as a locus involved in lipid metabolism in HF cows [82]. The *THRB* gene on BTA 27 was associated with FPR and RES-DV, and might be an interesting candidate gene when aiming for improvements for both traits, i.e., less susceptibility to *D. viviparus* infections and stable FPR, which has been already proven by May et al. [21] quantitative-genetically. Furthermore, we found seven pathways for genes associated with FPR (e.g., MAPK signaling pathway), which were associated with milk production and fat yield in different cattle breeds [62,83]. The overlap of pathways identified for fat yield in previous studies and FPR in our study might be explained by the fact that fat yield is more variable than protein yield, and thus, contributes to a larger variation in FPR.

For the health and metabolic stability indicator traits SCS and FPR, Jaeger [10] estimated genetic correlations lower than 0.80 between two classes of average herd sizes and first calving ages in DSN based on pedigree data, indicating the presence of genotype-by-environment interactions (GxE). Hence, we assume that a large number of genetic loci contribute to GxE for SCS and FPR, which should be explored in ongoing studies. The knowledge of how GxE contributes to the phenotypic variance for adaptive and characteristic traits in DSN is of great importance for the conservation of the breed, since large GxE SNP effects in cattle indicate considerable opportunity to improve environmental resistance and health [84]. For SCS, our GWAS revealed SVs on BTA 1, 9, 11, 13, 23, and 25. Significant marker associations for SCS in other dairy cattle breeds were described on each chromosome [85], indicating the complex genetic architecture of the trait. In the present study, we estimated additive genetic effects by neglecting non-additive dominance effects, which could play a crucial role in the genomic architecture of complex functional traits [86]. As pointed out by Howard et al. [87], dominance decreases when alleles are almost fixed due to inbreeding. Interestingly, a large inbreeding depression was observed for SCS in Holstein cows [88], while inbreeding depression for SCS is currently not present in DSN, possibly due to different selection strategies with only a small number of DSN bulls with high SCS breeding values used for artificial insemination [30]. Substantial dominance effects in DSN for endoparasite infection traits were estimated for an immunological relevant gene [89]. The largest number of associations was detected on BTA 25 within and close to the *RBFOX1* gene, which was previously associated with subclinical ketosis in Jersey cattle [90]. An interesting candidate gene associated with SCS was the *CLDN8* gene on BTA 1, which was found to be increasingly expressed in the bovine mammary glands during reduced milking frequency [91]. Furthermore, *CLDN8* is part of the cell adhesion molecules and leukocyte transendothelial migration pathways, being involved in the recruitment and activation of macrophages during acute *Escherichia coli*-induced mastitis [50,92]. Due to the biological relevance of *CLDN8* in udder health, we recommend a more detailed investigation of polymorphisms and favorable alleles in *CLDN8,* which might contribute to improved udder health in DSN. No overlap was seen between the genomic loci affecting SCS and loci affecting mastitis, which have been identified by Meier et al. [24]. In contrast to our findings for SCS, Meier et al. [25] detected genomic loci on BTA 3, 6, 9, and 26 for mastitis in a population of 1026 50K genotyped DSN, and they identified *BMPR1B* on BTA 6 as the main candidate gene associated with mastitis. The *BMPR1B* gene was described to be involved in immune response to the mastitis pathogen *Staphylococcus aureus* and is related to the cytokine–cytokine interaction and TGF-ß signaling pathway. In the present study, *BMPR1B* was associated with RES-DV assuming that *BMPR1B* plays an important role in both bacterial and endoparasite infections in DSN.

The GWAS revealed a large number of associations and candidate genes for endoparasite infection traits. Interestingly, we identified three genes associated with two endoparasite traits (*TRERF1* on BTA 23, *PIK3C3* on BTA 24, and *PCDH15* on BTA 26). The *PCDH15* gene, identified for GIN and *F. hepatica* infections, was associated with *D. viviparus* infections in our preliminary GWAS in DSN based on imputed high-density (HD) 700K data [22]. Hence, *PCDH15* seems to be involved in overall resistance against endoparasite infections in DSN. Twomey et al. [7] applied a GWAS for antibody responses to endoparasite infections (*F. hepatica*, *Neospora caninum*, and *Ostertagia ostertagi*) in dairy and beef cattle and identified up to 248 SVs and up to 48 QTL for the different traits, which is similar to the number of SNPs and candidate genes for endoparasite infection traits detected in our study. However, the number of detected SVs for fertility and health indicator traits was smaller, which might be due to smaller heritabilities or a more complex genetic architecture for genes involved in host immune response to parasitic disease. Another explanation for the larger number of SVs detected for endoparasite traits addresses the chosen pre-correction approach for endoparasite infection traits in order to consider a larger number of cows examined for endoparasite infections. However, when applying the same approach for fertility and health indicator traits and using the model residuals from pre-corrected traits, the number of significantly and suggestively associated markers did not increase substantially for these traits (results not shown). Furthermore, the genomic inflation factor λ ranged from 1014 to 1088 for all traits and pre-corrected endoparasite traits, indicating that the number of false-positive associations for endoparasite infection traits does not differ to the other investigated traits. 

For RES-GIN, a large number of associations surpassing *p*Bonf were detected on BTA 2, 8, 24, and 26. Using the same study design and statistical approach for endoparasite infection traits in DSN imputed to high-density (HD) 700K, we identified associations for RES-GIN on BTA 2, 4, 5, 8, 9, 18, 22, 24, and 26 in a previous GWAS [22]. Interestingly, a large proportion of SNPs detected with HD did not reach the significance threshold in the present study based on WGS data and only one candidate gene (*PHLPP1*) on BTA 24 overlapped in both datasets. The same observation was made by Wu et al. [6] when comparing GWAS results from different SNP panels and WGS data for the same trait. A possible explanation refers to differences in the genomic relationship matrix and LD between low- or high-density SNP panels and WGS data. Moreover, the significance thresholds in the present GWAS differs for different marker densities as a result of Bonferroni multiple testing correction and the calculated effective number of independent SVs. In consequence, markers detected with 700K HD data by May et al. [22] did not reach the suggestive and significance threshold in this study. However, for RES-DV, we detected linkage (*r*^2^ > 0.6) between 21 significantly associated SNPs from the GWAS by May et al. [22] and 258 significantly or suggestively associated SVs in the present study. Furthermore, when marker density increases, LD within a region becomes stronger, implying a larger number of SVs around the identified genes affecting the trait of interest. For fecal egg excretion in Angus cattle, Kim et al. [52] detected significant associations on BTA 3, 5, 8, 15, and 27 and, similar to our GWAS, they found genes annotated to the chemokine signaling and cytokine–cytokine interaction pathway. In contrast, Twomey et al. [7] identified the strongest associations on BTA 3, 4, 12, 13, 14, 19, 21, and 23 for antibody response to *O. ostertagi* (most common GIN species in cattle) in a multi-breed GWAS. We identified *ALPK2* on BTA 24 as the gene with the largest number of associations within and in close distance to the gene. Surprisingly, *ALPK2* is not involved in immunological pathways and was not reported as a gene underlying parasite resistance in other animal species. In accordance to the study by May et al. [22], the largest number and strongest associations for RES-GIN and RES-DV were detected on BTA 2 and on BTA 24, which might be explained by the biological relatedness of both nematode traits RES-GIN and RES-DV. However, we detected no overlapping gene for RES-GIN and RES-DV. The gene *FAM234B* on BTA 5 and the gene *DOK6* on BTA 24 were previously reported for *D. viviparus* larvae counts [22]. This is the first GWAS for patent *D. viviparus* infections in cattle based on WGS data and suggesting that the cytokine–cytokine interaction, JAK-STAT signaling, PI3K-signaling, and TGF-β signaling pathways are associated with *D. viviparus* infections in cattle.

For RES-FH, WGS data revealed a large number of candidate genes and related pathways previously described for *F. hepatica* infections via transcriptomic studies [51,53,56,59,63], indicating the advantage of WGS data to identify causal mutations for complex health traits in dairy cattle. We identified the fibroblast growth factor receptor 1 (*FGFR1*) gene on BTA 27 associated with RES-FH. Yu et al. [93] showed that fibroblast growth factor polypeptides are associated with liver fibrosis in mice. Liver fibrosis plays a key role in host immune response to *F. hepatica* infections [64]. Interestingly, TGF-ß plays a crucial role in fibrogenic processes during *F. hepatica* infections and acts as a host receptor for a parasitic growth factor [64,94]. Fu et al. [64] described the *SMAD4* gene on BTA 24 as a key factor involved in fibrogenetic processes during *F. hepatica* infections. Here, we detected *SMAD4* associated with RES-FH and related to the TGF-ß signaling pathway, indicating the power of our GWAS approach to identify genes associated with complex traits in DSN. Together with *IL21*, *SMAD4* is related to the Th17 cell differentiation pathway. Walsh et al. [66] showed that TGF-ß suppresses Th17 immune response in the host via *F. hepatica* infections. Furthermore, we detected six genes for RES-FH related to the cGMP-PKG signaling pathway and the JAK-STAT signaling pathway, which are well known to be involved in *F. hepatica* infections [56,59]. Surprisingly, the genes prolactin-related protein IIV and IX (*PLR9* and *PRP-VII*) on BTA 23 were associated with RES-FH and annotated to the JAK-STAT signaling pathway, too. This finding addresses possible polymorphisms in the identified prolactin genes in DSN associated with both improved milk production and resistance to *F. hepatica* infections. This is of great importance, since genetic correlations between milk production and *F. hepatica* egg excretion was shown to be favorable in quantitative-genetic studies [21,22]. Hence, genetic correlations with milk production traits should be taken into consideration when selecting DSN being well adapted to pasture production systems with improved fertility and metabolic stability and enhanced resistance to endoparasite and udder bacterial infections.

## 5. Conclusions

Using WGS data, we identified a large number of SVs and potential candidate genes associated with fertility, udder and metabolic health indicator traits, metabolic stability, and endoparasite infection traits in the local DSN population. Such in-depth insights into the genomic particularities enable improved selection strategies in the local DSN breed, especially when defining criteria in the context of conservation of genetic resources. The investigated traits are known as specific adaptive traits in DSN. Genetic improvements of these traits contribute to overall robustness and to possible DSN breed advantages or DSN breed competitiveness over large commercial dairy cattle populations. Selection of DSN animals carrying favorable alleles for the identified SVs is an efficient breeding approach in this regard. For CTFS and NR56, three annotated genes have similar functions in other cattle breeds. *CLDN8* and *RBFOX1* were the most interesting potential candidate genes for SCS. The largest number of associations for FPR were detected on BTA 12 and 27. For endoparasite infection traits, we detected potential candidate genes and related biological pathways, which are involved in host immune response to endoparasite infections. In particular, the identified markers within immunological relevant genes should be used for future genomic selection strategies in DSN, aiming to improve health and adaption to pasture environments.

## Figures and Tables

**Table 1:** Descriptive statistics for fertility traits, health and metabolic stability indicator traits, and endoparasite infection traits in DSN.

Trait ^1^	No. of Records	No. of Cows	No. of Cows with Sequence Level Genotypes	Mean ^2^	SD ^2^	Min. ^2^	Max. ^2^
CTFS	1683	1683	1683	78.31	27.44	26.0	241.0
NR56	1683	1683	1683	0.64	0.48	0	1.0
SCS	1638	1638	1638	2.58	1.48	−1.06	8.88
FPR	1638	1638	1638	1.18	0.18	0.45	2.42
FEC-GIN	1997	1166	142	11.35	22.57	0	225.0
FEC-FH	2006	1166	142	0.61	3.64	0	89.0
FLC-DV	1988	1163	142	0.17	2.14	0	46.0

^1^ CTFS = calving-to-first service interval; NR56 = non-return after day 56; SCS = somatic cell score (log-transformed somatic cell count: log2 (SCC/100,000) +3); FPR = fat-to-protein ratio; FEC-GIN = fecal egg count for gastrointestinal nematodes; FEC-FH = fecal egg count for *Fasciola hepatica*; FLC-DV = fecal larvae count for *Dictyocaulus viviparus*. ^2^ Mean, SD, Min. and Max. values are given for the number of records.

**Table 2:** Potential candidate genes related to the identified sequence variants (SVs) significantly associated with the female fertility traits calving-to first service interval (CTFS) and non-return 56 (NR56) in DSN.

BTA	Gene Position ^1^	No. of SVs within/Close to Gene ^2^	Position of Maximum Association (*p*-Value)	rs Number of Maximum Association	Gene Name
CTFS					
12	43,998,992–44,525,321	3/0	44,156,560 (2.93 × 10^−6^) *	rs136060929	*KLHL1*
13	25,463,655–25,592,062	5/10	25,577,261 (2.25 × 10^−7^) *	rs384930569	*ARHGAP21*
15	58,214,580–58,224,553	0/1	58,258,453 (1.32 × 10^−6^) *	rs41777070	*LIN7C*
	78,914,154–78,915,587	0/1	78,929,182 (2.08 × 10^−6^) *	rs379801720	*ENSBTAG00000052005*
	79,013,311–79,014,249	0/1	78,929,182 (2.08 × 10^−6^) *	rs379801720	*OR5BE5*
28	43,760,392–43,804,579	10/0	43,796,638 (1.37 × 10^−6^) *	rs42155599	*CHAT*
NR56					
8	96,644,114–96,759,752	2/0	96,724,190 (4.07 × 10^−7^) *	rs110809463	*ZNF462*
11	74,126,883–74,224,519	1/0	74,190,369 (8.30 × 10^−7^) *	rs383197946	*EFR3B*
20	56,953,217–57,071,806	0/1	56,867,118 (1.34 × 10^−6^) *	rs207515592	*MARCH11*

^1^ Gene position (start-end) in ENSEMBL build on *Bos taurus* genome assembly ARS-UCD1.2; ^2^ number of associations that reached the Bonferroni-corrected genome-wide significance threshold (*p*Bonf) or the suggestive significance threshold (*p*Sug) based on the position of the identified candidate gene ± 100 kb up- and downstream; * above *p*Sug; BTA = *Bos taurus* chromosome.

**Table 3:** Potential candidate genes related to the identified sequence variants (SVs) significantly associated with the udder health indicator trait somatic cell score (SCS) and with the metabolic health and stability indicator trait fat-to-protein ratio (FPR) in DSN.

BTA	Gene Position ^1^	No. of SVs within/Close to Gene ^2^	Position of Maximum Association (*p*-Value)	rs Number of Maximum Association	Gene Name
SCS					
1	5,689,903–5,690,709	0/1	5,693,321 (2.49 × 10^−6^) *	-	*KRTAP24-1*
	5,770,225–5,772,218	0/1	5,693,321 (2.49 × 10^−6^) *	-	*CLDN8*
9	15,242,718–15,373,861	1/0	15,301,365 (2.76 × 10^−6^) *	-	*SENP6*
11	101,323,401–101,414,345	0/1	101,435,611 (2.06 × 10^−6^) *	rs211669575	*NUP214*
	101,450,754–101,465,047	0/1	101,435,611 (2.06 × 10^−6^) *	rs211669575	*FAM78A*
	101,696,993–101,834,040	2/0	101,761,967 (1.30 × 10^−7^) **	rs137783421	*RAPGEF1*
13	61,874,277–61,955,381	0/1	61,959,686 (1.04 × 10^−6^) *	rs211178277	*NOL4L*
23	42,945,450–43,017,711	1/0	42,9749,92 (4.20 × 10^−7^) *	rs876215027	*RANBP9*
25	6,224,841–6,638,491	2/45	6,654,595 (2.58 × 10^−7^) *	rs136166815	*RBFOX1*
FPR					
9	83,287,300–83,713,833	1/0	83,557,515 (3.16 × 10^−7^) *	rs467161057	*GRM1*
11	6,481,883–6,555,244	0/4	6,464,004 (3.06 × 10^−7^) *	rs134892674	*ENSBTAG00000054755*
12	11,675,264–12,071,822	15/1	11,736,920 (1.39 × 10^−7^) **	rs439994366	*VWA8*
	12,256,326–12,294,096	0/1	12,195,520 (2.71 × 10^−6^) *	rs209487147	*ENSBTAG00000053271*
	12,410,713–12,461,551	0/9	12,496,948 (1.49 × 10^−6^) *	rs208793423	*AKAP11*
13	58,456,060–58,457,612	0/2	58,382,430 (2.12 × 10^−6^) *	rs42020993	*ENSBTAG00000054668*
	60,191,141–60,234,885	1/0	60,228,471 (2.65 × 10^−6^) *	-	*ANGPT4*
	61,107,684–61,147,486	2/0	61,145,994 (8.51 × 10^−7^) *	-	*HM13*
	62,381,695–62,408,299	0/1	62,434,039 (1.91 × 10^−6^) *	-	*BPIFB4*
	62,503,682–62,514,220	0/1	62,434,039 (1.91 × 10^−6^) *	-	*BPIFA2A*
	62,645,078–62,655,447	0/1	62,659,594 (2.82 × 10^−6^) *	-	*BPIFA2B*
	62,676,036–62,685,906	0/1	62,659,594 (2.82 × 10^−6^) *	-	*ENSBTAG00000031373*
	65,100,388–65,148,653	2/0	65,123,915 (1.50 × 10^−6^) *	rs109380861	*CNBD2*
	72,891,431–72,905,729	1/0	72,897,116 (1.46 × 10^−6^) *	rs137243257	*TTPAL*
	75,255,202–75,339,020	2/0	75,261,912 (2.42 × 10^−6^) *	rs137115876	*SLC13A3*
19	30,045,106–30,177,093	0/1	30,186,388 (2.15 × 10^−6^) *	-	*ENSBTAG00000049618*
27	36,454,819–36,470,324	0/12	36,520,069 (6.89 × 10^−7^) *	rs211250281	*GINS4*
	36,522,605–36,539,773	1/12	36,520,069 (6.89 × 10^−7^) *	rs211250281	*GPAT4*
	41,461,703–41,896,044	1/0	41,614,645 (2.30 × 10^−6^) *	rs135231909	*THRB*

^1^ Gene position (start-end) in ENSEMBL build on *Bos taurus* genome assembly ARS-UCD1.2; ^2^ number of associations that reached the Bonferroni-corrected genome-wide significance threshold (*p*Bonf) or the suggestive significance threshold (*p*Sug) based on the position of the identified candidate gene ± 100 kb up- and downstream; * above *p*Sug; ** above *p*Bonf; BTA = *Bos taurus* chromosome.

**Table 4:** Pathways (sorted alphabetically) related to the identified candidate genes associated with fat-to-protein ratio (FPR), somatic cell score (SCS), and endoparasite infection traits (RES-GIN, RES-FH, RES-DV), selected from the DAVID and KEGG databases and previously described in literature to be associated with the corresponding trait.

Pathway	KEGG Entry	Trait ^1^	Candidate Gene (BTA)	Possible Association of Pathway with Trait according to Literature
B cell receptor signaling pathway	bta04662	RES-GIN	*MALT1* (24)	Involvement of B cells in immune response to gastrointestinal nematodes in ruminants [48].
Calcium signaling pathway		FPR	*GRM1* (9)	Pathway associated with milk fat content in Holstein cattle [49].
Cell adhesion molecules	bta04514	SCS	*CLDN8* (1)	Identification of the cell adhesin molecules pathway for *Escherichia coli*-induced mastitis in RNAseq analysis [50].
		RES-DV	*CD226* (24)	Cell adhesion molecules pathway identified for *F. hepatica* infections in cattle [22].
cGMP-PKG signaling pathway	bta04022	RES-FH	*PLCB1* (13), *PRKG1* (26), *ADRB3* (27)	Cyclic GMP (cGMP) is an intracellular messenger that mediates the action of nitric oxide, which is increasingly produced by leukocytes during *F. hepatica* infections [51].
Chemokine signaling pathway	bta04062	RES-GIN	*ADCY1* (4)	Pathway was associated with resistance to gastrointestinal nematode infections in Angus cattle [52].
		RES-FH	*PLCB1* (13), *PTK2* (14)	Pathway was associated with *Fasciola gigantica* infections in Buffalo via proteomics analysis [53]; pathway associated with *F. hepatica* infections in mice [54].
C-type lectin receptor signaling pathway	bta04625	RES-GIN	*MALT1* (24)	C-type lectin receptors are involved in innate and adaptive immunity to pathogens [46]; helminth C-type lectins are involved in host–parasite interactions [55].
Cytokine–cytokine receptor interaction	bta04060	RES-GIN	*ACVR1C* (2)	Pathway was associated with resistance to GIN infections in Angus cattle [52] and in German Black Pied dairy cattle [22]; up- and downregulation of cytokines as immune mechanism in cattle in response to helminth infections [56,57].
		RES-DV	*BMPR1B* (6)	
		RES-FH	*IL21* (17), *PRP9* (23), *PRP-VII* (23)	
Estrogen signaling pathway	bta04915	RES-GIN	*ADCY1* (4)	Increase in reproduction rate of helminths as a result of increasing metabolism of 17-ß-estradiol in the host [58]
		RES-FH	*PLCB1* (13), *KRT31* (19), *KRT34* (19)	
JAK-STAT signaling pathway	bta04630	RES-FH	*IL21* (17), *PRP9* (23), *PRP-VII* (23)	*F. hepatica* excretory-secretory antigens suppress multiple proteins participating in the JAK-STAT signaling pathway in mice [59].
		RES-DV	*CCND3* (23), *SOCS6* (24)	JAK-STAT pathway is the principal signaling mechanism for cytokines involved in *D. viviparus* infections [46,60].
Leukocyte transendothelial migration	bta04670	RES-FH	*PTK2* (14)	Pathway was associated with *Fasciola gigantica* infections in buffalo via proteomics analysis [53].
		SCS	*CLDN8* (1)	Identification of the Leukocyte transendothelial migration pathway for *Escherichia coli*-induced mastitis in RNAseq analysis [50].
MAPK signaling pathway	bta04010	FPR	*ANGPT4* (13)	Pathway associated with milk fat traits in dairy cattle [61,62].
Natural killer cell mediated cytotoxicity	bta04650	RES-FH	*PTK2* (14)	Natural killer cells are lymphocytes of the innate immune response involved in host defense against infections with parasites [46]; cytotoxic natural killer cells were involved in early stage of infection by *F. hepatica* in rats [63]; pathway identified for *F. hepatica* infections in mice [54].
Neuroactive ligand-receptor interaction	bta04080	FPR	*GRM1* (9), *THRB* (27)	Pathway associated with milk fat traits in dairy cattle [49]
NF-kappa B signaling pathway	bta04064	RES-GIN	*MALT1* (24)	Family of transcription factors regulating genes involved in immunity [46]; pathway associated with *F. hepatica* infections in sheep [64].
NOD-like receptor signaling pathway	bta04621	RES-FH	*PLCB1* (13)	Family of pattern recognition receptors responsible for various pathogens and generating innate immune response [46].
Phospholipase D signaling pathway	bta04072	FPR	*GRM1* (9)	Phospholipase D is an essential enzyme for the production of phosphatidic acid, a key intermediate in milk fat synthesis during lactation [61].
PI3K-Akt signaling pathway	bta04151	RES-GIN	*PHLPP1* (24)	Pathway has important functions in cellular immune response [46].
		RES-FH	*PTK2* (14), *PRP9* (23), *PRP-VII* (23), *EIF4EBP1* (27), *FGFR1* (27)	
		RES-DV	*CCND3* (23), *LAMA3* (24)	
		FPR	*ANGPT4* (13)	Pathway associated with milk fat traits in dairy cattle [62].
Rap 1 signaling pathway	bta04015	FPR	*ANGPT4* (13)	Pathway associated with milk fat traits in dairy cattle [61].
Ras signaling pathway	bta04014	FPR	*ANGPT4* (13)	Pathway associated with milk fat traits in dairy cattle [61].
T cell receptor signaling pathway	bta04660	RES-GIN	*MALT1* (24)	Involvement of B cells in immune response to gastrointestinal nematodes in ruminants [48].
TGF-β signaling pathway	bta04350	RES-GIN	*ACVR1C* (2)	TGF-ß involved in host immune response during *Ostertagia ostertagi* (GIN species) infections [65]; pathway is associated in host–*F. hepatica* interactions: binding of *F. hepatica* growth factors to host TGF- ß receptors and triggering SMAD (Sma-and Mad-related proteins) in host leukocytes [56].
		RES-FH	*SMAD4* (24)	
		RES-DV	*BMPR1B* (6)	
Th17 cell differentiation	bta04659	RES-FH	*IL21* (17), *SMAD4* (4)	*F. hepatica*-induced TGF-ß suppresses Th17 responses in infected mice [66].

^1^ RES-GIN = residuals for fecal egg counts of gastrointestinal nematodes; RES-FH = residuals for fecal egg counts of liver flukes (*Fasciola hepatica*); RES-DV = residuals for fecal larvae counts of bovine lungworms (*Dictyocaulus viviparus*).

**Table 5:** Potential candidate genes (sorted alphabetically) related to the identified sequence variants (SVs) associated with residuals for fecal egg counts of gastrointestinal nematodes (RES-GIN), residuals for fecal egg counts of liver flukes (RES-FH), and residuals for fecal larvae counts of bovine lungworms (RES-DV) in DSN. Genes with at least one associated variant in the respective gene are marked in bold. A gene was considered a candidate gene if at least one SV above *p*Sug was positioned in the respective gene and/or within 100 kb up- and downstream of the gene.

BTA	RES-GIN	RES-FH	RES-DV
1	*ENSBTAG00000048985*	*-*	-
2	*ACVR1*, ***ACVR1C***, ***CNTNAP5***, *CPS1*, *ENSBTAG00000054211*, *ENSBTAG00000040367*, *ENSBTAG00000051630*, ***GPD2***, *KIF5C*, ***LANCL1***, ***LRP1B***, *LYPD6B*, ***MAP2***, *NR4A2*, ***UNC80***	***CRYGA***, *CRYGB*	*ENSBTAG00000033143* *, *ENSBTAG00000050185*, *ENSBTAG00000049959*, *ENSBTAG00000055116 FAM124B* *, ***GALNT13***, *GPR55* *, *SPATA3* *
3	*PDE4B*	***CDC14A***, *ENSBTAG00000037539*, ***KCNN3***, ***TTC4***	*-*
4	*ADCY1*	CNPY1*, *HTR5A* *, ***LMBR1***, *MNX1*, *NCAPG2* *, *PTPRN2* *, *RBM33* *, *UBE3C*	***ESYT2***
5	*-*	*-*	*ARHGDIB* *, *ASCL1*, *DRAM1*, ***EMP1*** *, ***FAM234B*** *, *GNPTAB*, ***GRIN2B*** *, ***LRP6***, ***NELL2***, *PDE6H* *, *SLC38A1*, *TPH2* *, ***UTP20***, *YAF2*, *ZCRB1*
6	***NDST4***	*GPRIN3*	***BMPR1B*** *, ***KCNIP4***, *PDLIM5* *
7	***MEGF10***	***ADAMTS19***, ***AADAT***, ***KIAA0825***, ***MAN2A1***, ***TMEM232*** *	*HAND1*
8	*ENSBTAG00000052065* *	*-*	*ALDH1A1*, *ENSBTAG00000052698*
9	*-*	***LCA5***, *SH3BGRL2*	*ENSBTAG00000055087* *, *SASH1* *, ***TBXT***, *UST* *
10	*-*	*ENSBTAG00000050159* *, ***TMEM87A***	*ABCD4*, *DDHD1*, *FERMT2*, *VRTN*
11	*-*	EXOC6B*, *FAM98A* *, REEP1*	*ENSBTAG00000009599*, ***LCN10***, ***NELFB***, ***RALGDS***, *TOR4A*
13	*-*	***PLCB1***, ***HAO1***	*SPINT3*
14	***CNBD1***	***PTK2***	***RALYL***
15	*-*	ARHGAP20*, ALX4*, *FAM111B* *, *GLYATL2* *	***CNTN5*** *
16	***SYT14***	*ENSBTAG00000053468*	*SOX13* *
17	*-*	*IL21*	*ENSBTAG00000033967*, *ENSBTAG00000055004*, *RAB36*, ***RSPH14***
18	***APRT***, ***CBFA2T3***, *DNAJA2*, ***GALNS***, *ENSBTAG00000048593*, *ENSBTAG00000048735*, *NETO2*, ***PHKB***	*-*	*-*
19	***HELZ***	*KRT31*, *KRT34*	
20	*-*	*-*	***DAB2***, *ENSBTAG00000047333*, *ENSBTAG00000000617*, ENSBTAG00000049964*
21	*-*	*-*	***APOPT1***, *ENSBTAG00000035184*, *ENSBTAG00000052298*, *ENSBTAG00000003957*, ***KLC1***, *MCTP2*, ***PPP1R13B***, *SNRPA1*, ***XRCC3***, ***ZFYVE21***
23		*ENSBTAG00000048946*, *ENSBTAG00000053227*, *KHDRBS2* *, *PRP9*, *PRP-VII*, ***TFAP2D***, ***TRERF1***	*CCND3*, ***FOXP4***, *LRFN2*, *TAF8*, *TRERF1*, *UBR2*
24	ALPK2*, *MALT1*, *NEDD4L* *, ***PHLPP1*****, *****TSHZ1***, ***ZCCHC2***, *ZNF532*	***ELAC1***, *MEX3C*, *PIK3C3*, *SMAD4*	***CD226*** *, ***CDH19***, *C24H18orf63*, ***CYB5A*** *, *DIPK1C*, ***DOK6*** *, ***DTNA*** *, *ENSBTAG00000049503*, ***LAMA3*** *, *NPC1*, ***OSBPL1A***, ***PIK3C3*** *, ***RTTN*** *, ***RIOK3*** *, *SOCS6* *, ***TMEM241***, *WDR7*
26	*BCL2*, ***CRTAC1*** *, *ENSBTAG00000048707*, *IDE* *, ***KIF11***, ***PCDH15***, *TNKS2*	***ATRNL1***, *EXOC6*, *HHEX*, ***PCDH15***, ***PRKG1***	*-*
27	*-*	*ADRB3*, *EIF4EBP1*, FGFR1*, *ZNF385D*	***THRB***
28	*-*	*ACTA1*, *ENSBTAG00000048654*	***ARHGAP22***
29	*-*	*PRMT3*, *SLC6A5*	*ENSBTAG00000008274*, *ENSBTAG00000050398*

* Genes including at least one associated variant above the genome-wide significance threshold (*p*Bonf = 1.22 × 10^−7^).

## Data Availability

All the data supporting the results of this article are included within the article. The raw phenotypic and genotypic data are stored in the databases of the IT company vit Verden and the cluster justHPC from Giessen University (https://www.hkhlr.de/de/cluster/justhpc-giessen, accessed on 2 June 2021). All data can be provided on request.

## Supplementary Materials

The following are available online at https://www.mdpi.com/article/10.3390/genes12081163/s1, Table S1: List of all SVs associated with calving-to-first-service interval (CTFS) and non-return at day 56 (NR56), Table S2: List of all SVs associated with somatic cell score (SCS) and fat-to-protein ratio (FPR), Table S3: List of all SVs associated with residuals of gastrointestinal nematode infections (RES-GIN), *Dictyocaulus viviparus* infections (RES-DV), and *Fasciola hepatica* infections (RES-FH), Table S4: Potential candidate genes with corresponding number of SVs within and close to the gene related to the identified SVs associated with residuals of fecal egg counts for gastrointestinal nematodes, Table S5: Potential candidate genes with corresponding number of SVs within and close to the gene related to the identified SVs associated with residuals of fecal egg counts for *Fasciola hepatica*, Table S6: Potential candidate genes with corresponding number of SVs within and close to the gene related to the identified SVs associated with residuals of fecal larvae counts for *Dictyocaulus viviparus.*
