# Supplementary material for: Genome-Wide Association Study Using Whole-Genome Sequence Data for Fertility, Health Indicator, and Endoparasite Infection Traits in German Black Pied Cattle
Source: Genes (Basel). 2021 Jul 28;12(8):1163. doi: 10.3390/genes12081163 (PMC8391191; doi:10.3390/genes12081163)
Supplement: Supplementary file 1 [file genes-12-01163-s001.zip › Table_S6.pdf]

Table S6. Potential candidate genes with corresponding number of sequence variants (SVs) within and close to the gene related to the identified SVs associated with residuals of fecal larvae counts for *Dictyocaulus viviparus*

BTA	Gene position	No. of SNP within gene / close to gene[2]	Position of maximum association (*P*-value)	SNP name of maximum association	Gene name
2	41,676,135 – 42,259,624	4/0	42,212,122 (1,90 x 10^{-7}) *	-	*GALNT13*
	43,416,111 – 43,416,743	0/1	43,351,774 (2.97 x 10^{-8}) **	rs378106986	*ENSBTAG00000033143*
	54,990,884 – 54,991,105	0/1	55,088,564 (8.12 x 10^{-7}) *	-	*ENSBTAG00000050185*
	112,489,090 – 112,502,288	0/14	112,466,120 (6.22 x 10^{-10}) *	rs135566755	*FAM124B*
	118,714,413 – 118,715,459	0/1	118,749,044 (8.29 x 10^{-8}) **	rs210011874	*GPR55*
	118,803,760 – 118,824,701	0/1	118,749,044 (8.29 x 10^{-8}) **	rs210011874	*SPATA3*
	132,076,996 – 132,079,294	0/2	132,093,680 (4.55 x 10^{-7}) *	rs478230560	*ENSBTAG00000049959*
	132,108,831 – 132,109,847	0/2	132,093,680 4.55 x 10^{-7}) *	rs478230560	*ENSBTAG00000055116*
4	119,432,686 – 119,508,052	2/0	119,489,954 (4.27 x 10^{-7}) *	rs384975315	*ESYT2*
5	1,542,142 – 1,644,590	0/6	1,738,107 (4.40 x 10^{-8}) **	rs381278803	*TPH2*
	33,940,564 – 33,993,305	0/4	34,077,374 (1.49 x 10^{-7}) *	rs110274733	*SLC38A1*
	35,475,234 – 35,861,464	2/0	35,722,296 (6.78 x 10^{-7}) *	rs440042874	*NELL2*
	65,183,368 – 65,269,247	1/0	65,227,455 (3.99 x 10^{-7}) *	rs210340351	*UTP20*
	65,570,868 – 65,655,965	0/4	65,687,745 (4.89 x 10^{-7}) *	rs209133559	*GNPTAB*
	65,703,174 – 65,738,912	0/4	65,687,745 (4.89 x 10^{-7}) *	rs209133559	*DRAM1*
	66,733,093 – 66,733,752	0/3	66,808,384 (3.39 x 10^{-7}) *	rs211113211	*ASCL1*
	94,923,693 – 94,321,648	0/6	94,943,346 (7.28 x 10^{-8}) **	rs440502252	*PDE6H*
	94,945,968 – 94,965,348	3/6	94,943,346 (7.28 x 10^{-8}) **	rs440502252	*ARHGDIB*

	95,970,070 – 96,313,866	2/28	96,379,458 (2.01 x 10^{-8}) **	rs380166151	*GRIN2B*
	96,633,068 – 96,654,177	1/21	96,497,709 (9.10 x 10^{-8}) **	rs378328839	*EMP1*
	96,766,338 – 96,785,806	0/8	96,710,031 (1.22 x 10^{-6}) *	rs210080551	*GSG1*
	96,786,665 – 96,862,648	1/0	96,813,852 (6.17 x 10^{-8}) **	-	*FAM234B*
	97,526,603 – 97,700,083	1/0	97,652,717 (2.26 x 10^{-7}) *	rs109416674	*LRP6*
6	29,373,117 – 29,593,444	1/1	29,632,595 (1.47 x 10^{-8}) **	rs210162448	*BMPR1B*
	29,911,322 – 30,142,653	0/2	29,858,997 (3.24 x 10^{-9}) **	-	*PDLIM5*
	40,250,033 – 41,576,485	2/0	41,434,831 (1.98 x 10^{-7}) *	-	*KCNIP4*
7	65,748,838 – 65,752,153	0/1	65,753,006 (5.24 x 10^{-7}) *	rs210704476	*HAND1*
8	48,982,548 – 49,003,364	0/1	49,035,376 (2.19 x 10^{-6}) *	rs209931492	*ENSBTAG00000052698*
	49,053,228 – 49,106,706	0/1	49,035,376 (2.19 x 10^{-6}) *	rs209931492	*ALDH1A1*
	70,279,848 – 70,315,972	0/1	70,307,273 (1.99 x 10^{-6}) *	rs458858907	*RHOBTB2*
9	3,049,813 – 3,050,755	0/72	3,016,828 (3.91 x 10^{-7}) **	-	*ENSBTAG00000055087*
	45,127,459 – 45,128,171	0/2	45,147,078 (6.76 x 10^{-7}) *	-	*ENSBTAG00000053468*
	86,010,125 – 86,327,460	0/2	85,981,007 (1.06 x 10^{-8}) **	rs208999925	*UST*
	101,115,142 – 101,160,469	1/4	101,114,103 (4.58 x 10^{-7}) *	rs209681677	*TBXT*
10	65,537,750 – 65,613,540	0/1	65,614,937 (3.32 x 10^{-7}) *	rs470290549	*FERMT2*
	65,673,444 – 65,741,258	0/1	65,614,937 (3.32 x 10^{-7}) *	rs470290549	*DDHD1*
	85,631,547 – 85,647,111	0/1	85,677,465 (1.88 x 10^{-6}) *	rs470290549	*ABCD4*
	85,679,086 – 85,681,188	0/1	85,677,465 (1.88 x 10^{-6}) *	rs470290549	*VRTN*
11	103,082,834 – 103,132,073	1/0	103,108,458 (5.06 x 10^{-7}) *	rs211549796	*RALGDS*
	105,841,421 – 105,854,755	0/1	105,857,287 (3.25 x 10^{-7}) *	rs384839378	*ENSBTAG00000009599*

	105,858,237 – 105,860,602	1/1	105,857,287 (3.25 x 10^{-7}) *	rs384839378	*LCN10*
	106,266,337 – 106,274,382	2/1	106,277,594 (2.86 x 10^{-7}) *	rs379503556	*NELFB*
	106,277,920 – 106,279,140	0/1	106,277,594 (2.86 x 10^{-7}) *	rs379503556	*TOR4A*
13	73,763,287 – 73,766,219	0/1	73,772,332 (2.26 x 10^{-6}) *	rs876078133	*SPINT3*
14	77,668,311 – 77,921,529	1/0	77,798,869 (3.91 x 10^{-7}) *	rs208878071	*RALYL*
15	8,786,754 – 9,437,352	47/56	8,742,915 (1.56 x 10^{-8}) **	rs41751445	*CNTN5*
16	1,899,514 – 1,911,011	0/1	1,823,258 (4.13 x 10^{-8}) **	rs211166165	*SOX13*
17	23,201,447 – 23,205,862	0/21	23,287,670 (1.29 x 10^{-7}) **	rs383287783	*ENSBTAG00000033967*
	39,268,470 – 39,268,712	0/1	39,354,863 (1.56 x 10^{-6}) *	rs459417776	*ENSBTAG00000055004*
	71,800,734 – 71,808,096	0/1	71,814,036 (1.50 x 10^{-6}) *	rs378582680	*RAB36*
	71,816,210 – 71,864,794	4/1	71,826,590 (1.92 x 10^{-7}) *	rs384409268	*RSPH14*
20	151,447 – 152,695	0/2	202,333 (1.40 x 10^{-7}) **	rs379281551	*ENSBTAG00000047333*
	217,375 – 218,720	0/2	202,333 (1.40 x 10^{-7}) **	rs379281551	*ENSBTAG00000000617*
	12,772,296 – 13,060,981	4/0	13,039,455 (9.68 x 10^{-8}) **	rs385171063	*ENSBTAG00000049964*
	35,003,821 – 35,062,931	1/3	35,059,415 (4.90 x 10^{-7}) *	rs41941680	*DAB2*
21	4,849,604 – 4,849,984	0/3	4,886,753 (1.50 x 10^{-6}) *	rs209637341	*ENSBTAG00000035184*
	4,945,634 – 5,195,923	0/3	4,886,753 (1.50 x 10^{-6}) *	rs209637341	*ENSBTAG00000052298*
	12,595,813 – 12,855,267	0/4	12,544,309 (2.36 x 10^{-7}) *	rs482879949	*MCTP2*
	29,153,369 – 29,166,386	0/1	29,190,236 (1.77 x 10^{-6}) *	rs211149344	*SNRPA1*
	29,231,423 – 29,236,481	0/1	29,190,236 (1.77 x 10^{-6}) *	rs211149344	*ENSBTAG00000003957*
	68,196,002 – 68,222,814	9/7	68,196,857 (9.67 x 10^{-7}) *	rs211385234	*APOPT1*
	68,243,678 – 68,304,998	59/7	68,299,895 (1.62 x 10^{-7}) *	-	*KLC1*

	69,308,102 – 68,317,607	4/0	68,315,294 (1.23 x 10^{-6}) *	rs210797928	*XRCC3*
	68,318,275 – 68,328,683	5/0	68,320,232 (4.41 x 10^{-7}) *	rs378142399	*ZFYVE21*
	68,329,806 – 68,412,242	4/0	68,346,802 (1.79 x 10^{-7}) *	rs211199721	*PPP1R13B*
23	14,301,055 – 14,502,729	0/2	14,562,162 (8.86 x 10^{-7}) *	-	*LRFN2*
	15,366,276 – 15,417,429	2/0	15,396,268 (9.29 x 10^{-7}) *	rs723725711	*FOXP4*
	15,698,661 – 15,705,476	0/1	15,781,973 (2.09 x 10^{-7}) *	rs383934845	*CCND3*
	15,794,648 – 15,814,397	0/1	15,781,973 (2.09 x 10^{-7}) *	rs383934845	*TAF8*
	15,939,656 – 16,145,761	0/1	16,226,142 (1.28 x 10^{-7}) **	-	*TRERF1*
	16,255,756 – 16,363,361	0/1	16,226,142 (1.28 x 10^{-7}) **	-	*UBR2*
24	4,042,759 – 4,060,302	0/1	4,096,809 (2.33 x 10^{-6}) *	rs432766648	*DIPK1C*
	4,103,783 – 4,127,824	0/1	4,096,809 (2.28 x 10^{-6}) *	rs432766648	*C24H18orf63*
	4,141,580 – 4,175,476	4/0	4,159,535 (2.62 x 10^{-10}) **	rs475726740	*CYB5A*
	7,020,091 – 7,021,668	0/21	6,939,507 (4.00 x 10^{-14}) **	rs470022980	*SOCS6*
	7,020,091 – 7,021,668	25/19	7,069,348 (4.75 x 10^{-12}) **	-	*RTTN*
	7,301,497 – 7,359,786	16/16	7,306,227 (5.38 x 10^{-12}) **	rs478657966	*CD226*
	7,406,095 – 7,815,350	52/94	7,814,348 (1.42 x 10^{-14}) **	rs137362837	*DOK6*
	10,135,849 – 10,168,160	1/0	10,163,946 (1.36 x 10^{-6}) *	rs385230524	*CDH19*
	13,877,432 – 14,042,408	7/0	13,956,760 (8.70 x 10^{-9}) **	rs379656891	*PIK3C3*
	22,130,833 – 22,449,938	1/0	22,194,507 (2.18 x 10^{-11}) **	-	*DTNA*
	32,306,070 – 32,535,370	1/0	32,306,779 (5.94 x 10^{-7}) *	rs42496419	*OSBPL1A*
	32,711,020 – 32,946,065	34/0	32,815,263 (5.36 x 10^{-8}) **	rs42606852	*LAMA3*
	33,058,613 – 33,105,720	3/0	33,088,536 (6.90 x 10^{-7}) *	rs207716226	*NPC1*

	33,143,190 – 33,165,434	1/0	33,151,533 (7.57 x 10^{-8}) **	rs207497553	*RIOK3*
	33,168,820 – 33,227,343	6/0	33,197,830 (2.36 x 10^{-7}) *	rs110248017	*TMEM241*
	51,460,887 – 51,464,511	0/7	51,363,633 (1.28 x 10^{-7}) **	rs77981186	*ENSBTAG00000049503*
	56,001,377 – 56,351,749	0/4	56,437,999 (5.63 x 10^{-7}) *	-	*WDR7*
	41,461,703 – 41,896,044	3/0	41,842,583 (1.60 x 10^{-7}) **	rs210015267	*THRB*
28	42,821,393 – 43,000,626	1/0	42,986,584 (1.84 x 10^{-6}) *	rs448566797	*ARHGAP22*
29	50,212,292 – 20,227,932	0/2	50,242,467 (1.69 x 10^{-6}) *	rs381115084	*ENSBTAG00000008274*
	50,263,520 – 50,284,468	0/2	50,242,467 (1.69 x 10^{-6}) *	rs381115084	*ENSBTAG00000050398*

[1] Gene position (start-end) in ENSEMBL build on assembly ARS 1.2; [2] Number of associations that reached the Bonferroni-corrected genome-wide significance threshold (*p*Bonf) or the suggestive chromosome-wide significance threshold (*p*Sug) based on the position of the identified candidate gene ± 100 kb up- and downstream; [3] Ensembl ID; *above *p*Sug; **above *p*Bonf; In case of several associations with the same *p*-value for one gene, the association with the lowest base pair position was presented
