# Supplementary material for: Genome-Wide Association Study Using Whole-Genome Sequence Data for Fertility, Health Indicator, and Endoparasite Infection Traits in German Black Pied Cattle
Source: Genes (Basel). 2021 Jul 28;12(8):1163. doi: 10.3390/genes12081163 (PMC8391191; doi:10.3390/genes12081163)
Supplement: Supplementary file 1 [file genes-12-01163-s001.zip › Table_S4.pdf]

Table S4. Potential candidate genes with corresponding number of sequence variants (SVs) within and close to the gene related to the identified SVs associated with residuals of fecal egg counts for gastrointestinal nematodes

BTA	Gene position	No. of SNP within / close to gene[2]	Position of maximum association (*P*-value)	SNP name of maximum association	Gene name
1	61,393,686 – 61,394,866	0/3	61,405,795 (1.66 x 10^{-6}) *	rs382795752	*ENSBTAG00000048985*
2	38,466,454 – 38,597,910	0/1	38,412,717 (7.10 x 10^{-7}) *	rs454395900	*ACVR1*
	38,753,355 – 38,831,144	2/0	38,803,764 (4.60 x 10^{-7}) *	rs384552992	*ACVR1C*
	39,370,481 – 39,375,220	0/5	39,289,095 (5.93 x 10^{-7}) *	-	*ENSBTAG00000054211*
	39,653,910 – 39,803,015	0/7	39,830,018 (9.87 x 10^{-7}) *	rs110034768	*GPD2*
	39,887,966 – 39,905,341	0/6	39,830,018 (9.87 x 10^{-7}) *	rs110034768	*NR4A2*
	46,966,296 – 47,080,898	0/3	47,170,597 (1.19 x 10^{-6}) *	-	*LYPD6B*
	47,212,782 – 47,368,935	0/3	47,170,597 (1.19 x 10^{-6}) *	-	*KIF5C*
	55,584,017 – 56,790,873	1/0	55,864,420 (7.60 x 10^{-7}) *	rs135503453	*LRP1B*
	76,113,318 – 76,921,405	24/0	76,234,116 (1.36 x 10^{-7}) **	rs42439607	*CNTNAP5*
	92,575,447 – 92,576,061	0/82	92,600,797 (1.85 x 10^{-7}) *	rs133440143	*ENSBTAG00000040367*
	92,793,751 – 92,797,284	0/24	92,778,941 (1.56 x 10^{-7}) *	rs133949764	*ENSBTAG00000051630*
	97,414,229 – 97,562,142	15/0	97,415,581 (2.36 x 10^{-6}) *	-	*MAP2*
	97,594,986 – 97,821,531	4/0	97,595,175 (2.36 x 10^{-6}) *	rs473257429	*UNC80*
	98,188,212 – 98,237,653	1/0	98,194,513 (2.40 x 10^{-6}) *	rs383165092	*LANCL1*
	98,467,680 – 98,467,680	1/0	98,513,660 (2.08 x 10^{-6}) *	rs385100711	*CPS1*
3	78,957,184 – 79,406,648	0/2	78,920,007 (8.47 x 10^{-7}) *	rs461282126	*PDE4B*
6	10,482,289 – 10,799,267	1/0	10,559,212 (9.18 x 10^{-7}) *	rs209360745	*NDST4*

7	26,554,784 – 26,762,374	1/0	26,748,138 (2.09 x 10^{-6}) *	rs208041628	*MEGF10*
8	12,514,670 – 12,517,391	2/7	12,511,433 (3.83 x 10^{-9}) **	rs133302281	*ENSBTAG00000052065*
14	75,334,895 – 75,781,689	8/0	75,689,630 (3.44 x 10^{-7}) *	rs1118060283	*CNBD1*
16	73,169,607 – 73,359,804	3/0	73,237,178 (1.90 x 10^{-6}) *	rs381568836	*SYT14*
18	14,013,051 – 14,015,824	1/1	14,015,023 (2.42 x 10^{-6}) *	rs448288990	*APRT*
	14,016,352 – 14,032,348	1/1	14,028,019 (2.42 x 10^{-6}) *	rs480067465	*GALNS*
	14,051,588 – 14,127,897	2/0	14,104,757 (2.77 x 10^{-7}) *	rs109237628	*CBFA2T3*
	15,375,166 - 15,386,724	0/1	15,444,409 (9.21 x 10^{-7}) *	rs458296509	*DNAJA2*
	15,485,075 – 15,548,896	0/1	15,444,409 (9.21 x 10^{-7}) *	rs458296509	*NETO2*
	15,865,422 – 16,091,810	1/0	15,963,389 (1.50 x 10^{-6}) *	rs441101196	*PHKB*
	63,606,513 – 63,606,851	0/1	63,653,699 (3.54 x 10^{-7}) *	-	*ENSBTAG00000048593*
	63,655,336 – 63,655,674	0/1	63,653,699 (3.54 x 10^{-7}) *	-	*ENSBTAG00000048735*
19	63,147,954 – 63,292,314	2/0	63,202,679 (7.08 x 10^{-7}) *	rs109598587	*HELZ*
24	3,403,848 – 3,482,982	1/0	3,414,842 (7.00 x 10^{-7}) *	rs383932767	*TSHZ1*
	57,348,965 – 57,546,293	0/30	57,609,548 (3.09 x 10^{-8}) **	rs210974887	*NEDD4L*
	57,611,636 – 57,740,561	23/58	57,614,785 (6.62 x 10^{-9}) **	rs437851546	*ALPK2*
	57,775,162 – 57,839,220	0/29	57,747,818 (7.66 x 10^{-7}) *	-	*MALT1*
	57,933,911 – 58,044,908	2/2	57,966,602 (7.66 x 10^{-7}) *	rs381385335	*ZNF532*
	60,903,462 – 60,958,117	1/0	60,949,698 (1.39 x 10^{-6}) *	rs380426784	*ZCCHC2*
	61,054,481 – 61,278,037	3/1	61,231,875 (4.25 x 10^{-7}) *	rs524661804	*PHLPP1*
	61,396,513 – 61,588,241	0/1	61,231,875 (4.25 x 10^{-7}) *	rs524661804	*BLC2*
26	4,657,603 – 5,569,857	1/0	5,249,642 (2.06 x 10^{-6}) *	rs137644536	*PCDH15*

13,347,972 – 13,411,176	0/2	13,421,458 (1.02 x 10^{-6}) *	rs461716772	*TNKS2*
13,454,223 – 13,454,223	0/2	13,421,458 (1.02 x 10^{-6}) *	rs461716772	*ENSBTAG00000048707*
13,881,642 – 13,976,189	0/19	13,981,029 (6.51 x 10^{-8}) **	rs459593018	*IDE*
14,004,835 – 14,050,631	8/19	13,981,029 (6.51 x 10^{-8}) **	rs459593018	*KIF11*
19,019,143 – 19,178,819	10/0	19,075,353 (8.83 x 10^{-8}) **	rs478372469	*CRTAC1*

[1] Gene position (start-end) in ENSEMBL build on assembly ARS 1.2; [2] Number of associations that reached the Bonferroni-corrected genome-wide significance threshold (*p*Bonf) or the suggestive chromosome-wide significance threshold (*p*Sug) based on the position of the identified candidate gene ± 100 kb up- and downstream; [3] Ensembl ID; *above *p*Sug; **above *p*Bonf; In case of several associations with the same *p*-value for one gene, the association with the lowest base pair position was presented
